# Prophylactic use of anti-emetic medications reduced nausea and vomiting associated with exenatide treatment: a retrospective analysis of an open-label, parallel-group, single-dose study in healthy subjects

**DOI:** 10.1111/j.1464-5491.2010.03085.x

**Published:** 2010-10

**Authors:** C Ellero, J Han, S Bhavsar, B B Cirincione, M B DeYoung, A L Gray, I Yushmanova, P W Anderson

**Affiliations:** Amylin Pharmaceuticals Inc.San Diego, CA; * Lilly USA, LLCIndianapolis, IN, USA

**Keywords:** anti-emetics, exenatide, nausea, vomiting

## Abstract

**Aims:**

Transient nausea and, to a lesser extent, vomiting are common adverse effects of exenatide that can be mitigated by dose titration and usually do not result in treatment discontinuation. This retrospective analysis of data from a phase 1, open-label, parallel-group, single-dose study in healthy subjects evaluated the effect of oral anti-emetics on exenatide-associated nausea and vomiting and on the pharmacokinetics of exenatide.

**Methods:**

A single subcutaneous dose (10 μg) of exenatide was administered to 120 healthy subjects (19–65 years, BMI 23–35 kg/m^2^). Incidences of nausea and vomiting were compared between 60 subjects premedicated with two oral anti-emetics 30 min before the exenatide dose and 60 non-premedicated subjects. Similarly, the area under the concentration-time curve (AUC) and the maximum observed concentration (C_max_) of plasma exenatide concentrations over 8 h post-dose were compared.

**Results:**

Among all subjects [61% male, 32 ± 12 years, body mass index (BMI) 29.1 ± 3.4 kg/m^2^ (mean ± sd)], mild to moderate nausea was the most frequent adverse event after exenatide dosing. Vomiting was also observed. Subjects premedicated with anti-emetics experienced significantly less nausea and vomiting (16.7 and 6.7%, respectively) vs. non-premedicated subjects (61.7 and 38.3%, respectively; *P*-value < 0.0001 for both nausea and vomiting). The mean area under the concentration-time curve and the maximum observed concentration AUC and C_max_ of plasma exenatide concentrations during 8 h post-dose were not significantly different between groups.

**Conclusion:**

Administration of oral anti-emetics before a single 10-μg exenatide dose was associated with significant reductions in treatment-emergent nausea and vomiting, with no discernible effect on the pharmacokinetics of exenatide. Use of anti-emetic therapy may provide a short-term strategy to minimize the nausea and vomiting associated with exenatide treatment.

## Introduction

Exenatide, a first-in-class glucagon-like peptide 1 (GLP-1) receptor agonist, is approved as an adjunct to diet and exercise and/or to some oral anti-diabetic therapies for the treatment of Type 2 diabetes mellitus. Exenatide improves glycaemic control by enhancing glucose-dependent insulin secretion by pancreatic B-cells, suppressing inappropriately elevated glucagon secretion, slowing gastric emptying, and reducing food intake [1]. Exenatide is administered as a subcutaneous injection at a starting dose of 5 μg twice daily (b.i.d.) which can be increased to 10 μg b.i.d.

The most commonly reported adverse event associated with exenatide b.i.d. treatment is nausea, which in five placebo-controlled registration trials in patients with Type 2 diabetes had an incidence ranging from 8 to 44% in exenatide-treated patients (vs. 0 to 18% for placebo) [1–6]. Vomiting was also observed, with an incidence of 4 to 13% (vs. 0 to 4% for placebo) [1–6]. In these trials, less nausea and vomiting were observed in patients treated only with exenatide than in patients treated with exenatide and oral anti-diabetic therapies (8 vs. 44% for nausea and 4 vs. 13% for vomiting, respectively) [1–6]. The nausea observed was generally of mild to moderate intensity, had a higher incidence during the first 8 weeks of therapy and then decreased over time with continued therapy [1,3–6]. The nausea associated with exenatide therapy is thought to be the result of slowed gastric emptying, appetite suppression and stimulation of neural GLP-1 receptors.

Strategies have been investigated to reduce nausea and vomiting. Gradual dose escalation was shown to be effective in mitigating nausea and vomiting in an earlier study [7] and was therefore used in the development of the treatment protocol for exenatide (5 μg for the first month, followed by 10 μg). However, nausea and, to a lesser extent, vomiting remained among the most commonly reported treatment-emergent adverse events in the five registration trials, although withdrawals from treatment due to these side effects were generally low (from 0.6 to 4% in four trials and 9% in one trial) [1–6]. There is also anecdotal evidence for the effectiveness of other strategies implemented by treating physicians to minimize nausea associated with exenatide treatment (e.g. reducing food portion size) [8]. Furthermore, it has been hypothesized that short-term use of anti-emetic medications may be effective in preventing nausea.

This manuscript reports the results of a retrospective analysis of data from a phase 1, open-label, parallel-group, single-dose, pharmacokinetic and safety study conducted in healthy subjects that included the use of anti-emetic medications in the protocol. While the study utilized anti-emetics primarily for subject retention purposes, because the study medication was not titrated, this *post hoc* analysis was performed to evaluate their effectiveness in mitigating the nausea and vomiting associated with exenatide treatment and their effect on the pharmacokinetics of exenatide.

## Subjects and methods

### Subjects

Subjects were eligible for inclusion in the study if they were males or non-pregnant, non-lactating females; age 19–65 years; and normal weight, overweight or obese, but otherwise healthy (BMI 23–35 kg/m^2^). Main exclusion criteria included: a history of diabetes mellitus; current hypertension; cardiovascular, hepatic, renal, gastrointestinal and central nervous system disease; and previous exposure to exenatide.

### Study design and interventions

The study was conducted in 120 subjects enrolled at a single clinical study site in the USA between March and September 2008. The study protocol was approved by the local Institutional Review Board and all subjects provided written informed consent. The study was conducted in accordance with the principles described in the Declaration of Helsinki (1964) and subsequent amendments.

As part of the study, a single subcutaneous injection of 10 μg exenatide was administered to all subjects. Blood samples were collected over 8 h following administration of the exenatide dose to measure plasma exenatide concentrations. Safety was assessed throughout the study by examination of data for adverse events, clinical laboratory values, vital signs and physical examinations. The assessment period following the 10-μg exenatide dose was approximately 1 day.

A higher incidence of nausea was anticipated (compared with previous studies) because the single dose of study medication (10 μg) was twice the recommended starting dose of exenatide. Hence, the protocol allowed administration of up to two prophylactic oral anti-emetics, at the discretion of the investigator, approximately 30 min prior to the exenatide injection in a subgroup of 60 subjects. All subjects in this subgroup received both metoclopramide (Reglan® Alaven Pharmaceutical LLC, Marietta, GA, USA, 10 mg) and ondan-setron hydrochloride (Zofran® Glaxo-SmithKline, Research Triangle Park, NC, USA, 8 mg).

### Statistical analyses

The intent-to-treat population was defined as all enrolled subjects who received at least one dose of study medication. Adverse events were coded using the Medical Dictionary for Regulatory Activities (MedDRA®) version 11.0 (http://www.meddramsso.com). Treatment-emergent adverse events were defined as those events that occurred for the first time or existed prior to and worsened during the 1 day following the exenatide dose. Treatment-emergent adverse events were summarized descriptively for the intent-to-treat population by Preferred Term, as defined by MedDRA and intensity. The intensity of each adverse event was characterized as ‘mild’ if the adverse event was transient, required no special treatment and did not interfere with the subject's daily activities; ‘moderate’ if the adverse event caused a low level of inconvenience to the subject, may have interfered with daily activities, but was ameliorated by simple therapeutic measures; or ‘severe’ if the adverse event interrupted a subject's daily activities and required systemic drug therapy or other treatment. The pharmacokinetic parameters area under the concentration-time curve from time zero to time of last quantifiable sample [AUC_(0–tlast)_] and maximum observed concentration from time zero to time of last quantifiable sample [C_max(0–tlast)_] of plasma exenatide concentrations over 8 h following exenatide dosing were determined using the non-compartmental method and summarized descriptively for intent-to-treat subjects with valid exenatide pharmacokinetic profiles by anti-emetic treatment group (premedicated and non-premedicated subjects).

A *post hoc* analysis was performed to determine whether there were statistically significant differences in the incidences of treatment-emergent nausea and vomiting between subjects who did and did not receive anti-emetics. *P*-values were calculated using the Fisher Exact test. Similarly, the pharmacokinetic parameters AUC_(0–tlast)_ and C_max(0–tlast)_ of plasma exenatide concentrations over 8 h post-dose were compared between groups to evaluate the potential effects of the anti-emetics on the pharmacokinetics of exenatide. *P*-values were calculated using Student's *t*-test.

## Results

### Baseline characteristics and subject disposition

All 120 enrolled subjects were included in the intent-to-treat population. Subjects were: 78% Caucasian, 9% Black, 8% Hispanic, 3% Native American, 1% Asian and 2% other ethnicities; 61% male; age 32 ± 12 years, with a screening BMI of 29.1 ± 3.4 kg/m^2^ (mean ± sd). Demographic and baseline characteristics in subjects who did and did not receive anti-emetics were comparable.

### Nausea and vomiting adverse events

**FIGURE 1:**
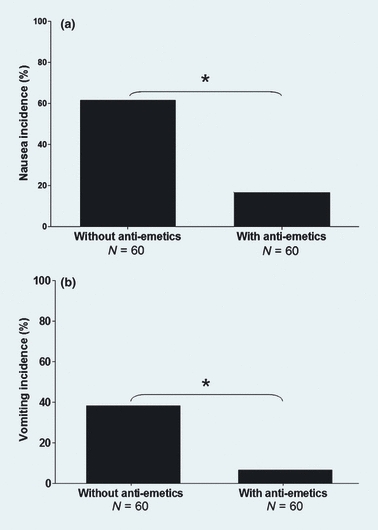
Incidence of nausea (a) and vomiting (b) during approximately 1 day after a single subcutaneous exenatide dose (10 μg) in subjects non-premedicated and premedicated with anti-emetics. Data are for the intent-to-treat population (*N* = 120). Incidence is the percentage of subjects experiencing the adverse event based on the number of intent-to-treat subjects in each group. Subjects experiencing multiple episodes of a given adverse event are counted once. *P < 0.0001 for premedicated vs. non-premedicated subjects.

The adverse event profile during the exenatide assessment period was similar to that observed in previous studies with exenatide. Nausea was the most frequent adverse event reported after the exenatide dose, with 39.2% of subjects experiencing at least one episode; vomiting occurred in 22.5% of subjects (Table 1). Both nausea and vomiting adverse events were of mild to moderate intensity (Table 1). As expected, a higher incidence of nausea and vomiting was observed in subjects who had not received anti-emetics, consistent with the introduction of exenatide at a dose (10 μg) higher than the recommended starting dose. This group had higher incidences of nausea and vomiting (61.7 and 38.3%, respectively) compared with subjects premedicated with anti-emetics (16.7 and 6.7%, respectively) (Table 1 and Fig. 1). The differences in the incidences of nausea (45%) and vomiting (31.6%) between premedicated subjects and non-premedicated subjects were statistically significant (*P*-value < 0.0001 for both nausea and vomiting) (Fig. 1).

**Table 1:** Treatment-emergent nausea and vomiting adverse events occurring during approximately 1 day following a single subcutaneous exenatide dose (10 μg) by MedDRA Preferred Term, intensity and anti-emetic treatment [population: ITT (*n* = 120)]

	Group by anti-emetic treatment								
	
	Non-premedicated (*n* = 60)*			Premedicated (*n* = 60)*			All subjects (*n* = 120)*		
			
Preferred term Intensity	*n*†	(%)	Events	*n*†	(%)	Events	*n*†	(%)	Events
Nausea	37	(61.7)	41	10	(16.7)	12	47	(39.2)	53
Mild	35	(58.3)	36	8	(13.3)	10	43	(35.8)	46
Moderate	5	(8.3)	5	2	(3.3)	2	7	(5.8)	7
Severe	0	(0.0)	0	0	(0.0)	0	0	(0.0)	0
Vomiting	23	(38.3)	47	4	(6.7)	5	27	(22.5)	52
Mild	23	(38.3)	36	4	(6.7)	5	27	(22.5)	41
Moderate	2	(3.3)	11	0	(0.0)	0	2	(1.7)	11
Severe	0	(0.0)	0	0	(0.0)	0	0	(0.0)	0

* Number of ITT subjects in the corresponding group.

† Number of subjects who experienced the adverse event.

Percentages are based on the number of ITT subjects in each group or in ‘All subjects’. Subjects experiencing multiple episodes of a given adverse event are counted once.

ITT, intent to treat; MedDRA®, Medical Dictionary for Regulatory Activities version 11.

In the group that did not receive anti-emetics, 21 of the 37 subjects who experienced nausea also vomited; in the group premedicated with anti-emetics, three of the 10 subjects who experienced nausea also vomited. In addition to receiving prophylactic anti-emetic medications prior to the exenatide dose, one of the 60 premedicated subjects was administered a second dose (4 mg) of ondansetron hydrochloride on the day of the exenatide dose to treat treatment-emergent nausea. Likewise, one subject in the non-premedicated group was given one dose (4 mg) of ondansetron hydrochloride to treat treatment-emergent vomiting.

A higher incidence of somnolence was reported in the group premedicated with anti-emetics (8.3%) compared with the non-premedicated group (1.7%) (data not shown). Somnolence has been reported with exenatide treatment post-approval [1] and had an incidence of 1% in the three registration trials of exenatide in combination with oral anti-diabetic therapy (30 weeks each) and up to 6% in other studies of exenatide [including short-term (28 days), uncontrolled and clinical pharmacology studies; data not shown]. Furthermore, drowsiness is a reported adverse effect of both metoclopramide and ondansetron [9,10].

### Plasma exenatide concentrations

Of the 120 intent-to-treat subjects, four subjects in the group premedicated with anti-emetics were excluded from the pharmacokinetic analysis because of incomplete pharmaco-kinetic profiles (i.e. less than three quantifiable post-dose measurements), leaving a total of 116 intent-to-treat subjects evaluable for pharmacokinetic analysis.

There were no statistically significant differences in geometric mean AUC and C_max_ of plasma exenatide concentrations during the 8 h following exenatide dosing between subjects who did and did not receive anti-emetics (Table 2), indicating that the administration of anti-emetic medications had no discernible effect on the pharmacokinetics of exenatide.

**Table 2:** Pharmacokinetic parameters of plasma exenatide concentrations following a single subcutaneous exenatide dose (10 μg) by anti-emetic treatment [population: ITT subjects evaluable for pharmacokinetic analysis (*n* = 116)]

	Group by anti-emetic treatment		
		
	Non-premedicated (*n* = 60)	Premedicated (*n* = 56)	*P*-value
Geometric mean AUC_(0–tlast)_ (se) (pg × h/ml)	863.53 (30.540)	818.38 (38.065)	0.36
Minimum, maximum	556.6, 1763.3	191.1, 1511.1	
Geometric mean C_max(0–tlast)_ (se) (pg/ml)	242.74 (12.404)	227.36 (15.361)	0.44
Minimum, maximum	109.1, 705.4	92.6, 906.9	

Geometric mean = exp{mean[log(X)]}; se of geometric mean = geometric mean × se of mean[log(X)].

AUC_(0–tlast)_, area under the concentration-time curve calculated using the linear trapezoidal method from time zero to tlast; C_max(0–tlast),_ maximum concentration observed during the blood sampling period from time zero to time tlast; ITT, intent to treat; tlast, time of last quantifiable sample during the 8-h post-dose collection period. SE, standard error.

## Discussion

This retrospective analysis demonstrated that prophylactic use of two oral anti-emetic medications led to statistically and clinically significant reductions in nausea and vomiting associated with exenatide administered as a single 10-μg dose to healthy subjects. The analysis also indicated that concomitant use of anti-emetics did not alter the pharmacokinetic properties of exenatide, suggesting that the differences in the incidences of nausea and vomiting between subjects premedicated and non-premedicated with anti-emetics were not attributable to differences in plasma exenatide concentrations in the two groups.

The results of this analysis represent the first published data demonstrating the effectiveness of oral anti-emetic medications in mitigating exenatide-induced nausea and vomiting. Consistent with previous exenatide trials, nausea and vomiting were frequently reported adverse events in this study, with incidences in subjects non-premedicated with anti-emetics (61.7 and 38.3% for nausea and vomiting, respectively) higher than previously observed in studies of exenatide monotherapy or in combination with anti-diabetic therapy (8–44% for nausea and 4–13% for vomiting) [1], all of which employed dose titration from 5 to 10 μg b.i.d. The current analysis indicates that prophylactic oral anti-emetics led to robust reductions in nausea and vomiting in the premedicated group following introduction of exenatide, even at double the recommended starting dose.

Several limitations of the study design and current analysis warrant consideration with regard to extrapolating these results beyond the context of this study. The study used a single, fixed dose of exenatide, with oral anti-emetics administered at a prespecified time (30 min prior to dosing). Therefore, the study did not address the effect of repeated administration of anti-emetics with continued exenatide treatment or the optimal time of anti-emetic administration relative to exenatide dosing. Additionally, all subjects in the premedicated group received both metoclopramide and ondansetron hydrochloride. It is not known whether administration of either anti-emetic agent alone would suffice in mitigating nausea and/or vomiting. (Although metoclopramide and ondansetron hydrochloride are not generally indicated for the treatment of medication-induced nausea, they were selected because both are commonly used in clinical practice for this purpose.) Additional potential limitations of the study design include the subject population, which comprised healthy individuals whose tolerance to exenatide may be different from that in patients with diabetes, and the open-label trial design, which inherently invites the potential for bias because it can affect patient expectations. Notwithstanding the aforementioned limitations, the results of this analysis provide evidence that prophylactic oral anti-emetics significantly reduced nausea and vomiting following administration of exenatide and may be relevant to clinical practice.

Implementing strategies to address the potential nausea and vomiting adverse events associated with exenatide treatment could promote patient's adherence to the treatment regimen and facilitate the achievement of glycaemic targets [11]. Exenatide is an effective glucoregulatory agent that also enhances satiety, leading to reduction in food intake and weight loss [1,12], an effect that can improve glycaemic control in patients with Type 2 diabetes [12]. Exenatide treatment (5 or 10 μg twice daily) b.i.d.) for up to 3 years as an adjunct to either diet and exercise or to oral anti-diabetic therapies in patients with Type 2 diabetes who were suboptimally controlled on such regimens produced significant reductions from baseline to study end in HbA_1c_, fasting plasma/serum glucose concentrations, body weight (all of which were sustained over 3 years) and postprandial plasma glucose concentrations [1–6,12], as well as improvements in cardiovascular risk factors (blood pressure, high-density lipoprotein and low-density lipoprotein cholesterol and triglycerides) and liver injury biomarkers (alanine transaminase and aspartate aminotransferase), although further investigation of the latter effects is warranted [12].

Gradual dose escalation at initiation of exenatide therapy is recommended, having been shown to be effective at mitigating nausea and vomiting with no loss of glucoregulatory activity [7]. In clinical practice, exenatide is initiated at 5 μg b.i.d. and can be increased after one month to 10 μg b.i.d., to minimize gastrointestinal adverse events.

There is also anecdotal evidence of additional strategies that have been employed to reduce nausea associated with exenatide [8]. These include education of patients treated with exenatide on the potential gastrointestinal side effects, including nausea and sensations of fullness, based on the observation that patients are better able to manage the nausea if they are forewarned that it might occur initially, might worsen during dose titration and should gradually subside with continued therapy. Other methods to ease nausea suggested by diabetes educators include: reduction in food portion size at mealtime to help address the sensation of fullness and reduce the risk of nausea, given the suspicion that the sensation reported in clinical trials as nausea may have been a sensation of fullness for some individuals; administering the exenatide injection just before a meal within the recommended 1-h window prior to meals; increasing fluid intake; symptomatically using antacids; and consuming diet or sugar-free carbonated beverages [8].

Although nausea is mostly of mild to moderate intensity and its incidence and intensity generally decrease after the first 8 weeks of exenatide treatment [1,3–6], prophylactic use of anti-emetics may provide an additional short-term strategy for the management of nausea at treatment initiation, during dose escalation and the first weeks of treatment, or in patients who continue to experience nausea over time. Additional studies are warranted to investigate the effectiveness of individual anti-emetic medications on nausea and vomiting with exenatide treatment, their ideal timing of administration relative to exenatide dosing, how long to administer these medications during continued treatment with exenatide and the safety of longer-term treatment with anti-emetics.

## Abbreviations

AUC: area under the concentration-time curve
BMI: body masss index
C_max_: maximum observed concentration
GLP-1: glucagon-like peptide 1
HDL: high-density lipoprotein cholesterol
LDL: low-density ipoprotein cholesterol
MedDRA®: Medical Dictionary for Regulatory Activities
SD: standard deviation
SE: standard error
tlast: time of last quantifiable sample

## References

[1] Amylin Pharmaceuticals Inc (2009). BYETTA® exenatide injection (prescribing information).

[2] Moretto TJ, Milton DR, Ridge TD, Macconell LA, Okerson T, Wolka AM (2008). Efficacy and tolerability of exenatide monotherapy over 24 weeks in antidiabetic drug-naive patients with type 2 diabetes: a randomized, double-blind, placebo-controlled, parallel-group study. Clin Ther.

[3] Kendall DM, Riddle MC, Rosenstock J, Zhuang D, Kim DD, Fineman MS (2005). Effects of exenatide (exendin-4) on glycemic control over 30 weeks in patients with type 2 diabetes treated with metformin and a sulfonylurea. Diabetes Care.

[4] Buse JB, Henry RR, Han J, Kim DD, Fineman MS, Baron AD (2004). Effects of exenatide (exendin-4) on glycemic control over 30 weeks in sulfonylurea-treated patients with type 2 diabetes. Diabetes Care.

[5] DeFronzo RA, Ratner RE, Han J, Kim DD, Fineman MS, Baron AD (2005). Effects of exenatide (exendin-4) on glycemic control and weight over 30 weeks in metformin-treated patients with type 2 diabetes. Diabetes Care.

[6] Zinman B, Hoogwerf BJ, Durán García S, Milton DR, Giaconia JM, Kim DD (2007). The effect of adding exenatide to a thiazolidinedione in suboptimally controlled type 2 diabetes: a randomized trial. Ann Intern Med.

[7] Fineman MS, Shen LZ, Taylor K, Kim DD, Baron AD (2004). Effectiveness of progressive dose-escalation of exenatide (exendin-4) in reducing dose-limiting side effects in subjects with type 2 diabetes. Diabetes Metab Res Rev.

[8] Peters AL, Miller D (2007). Case study 2: new insights: clinical pearls for using incretin mimetics in type 2 diabetes. Diabetes Educ.

[9] Alaven Pharmaceutical LLC (2009). Reglan® metoclopramide hydrochloride tablets (prescribing information).

[10] GlaxoSmithKline (2009). Zofran (ondansetron hydrochloride) tablets (prescribing information).

[11] Siminerio L (2006). Challenges and strategies for moving patients to injectable medications. Diabetes Educ.

[12] Klonoff DC, Buse JB, Nielsen LL, Guan X, Bowlus CL, Holcombe JH (2008). Exenatide effects on diabetes, obesity, cardiovascular risk factors and hepatic biomarkers in patients with type 2 diabetes treated for at least 3 years. Current Medical Research & Opinion.

